# Fetal urinary ascites caused by bladder rupture due to paraurethral cyst: a rare case

**DOI:** 10.1007/s00404-026-08415-1

**Published:** 2026-04-13

**Authors:** Marian Pekar Zlotin, Orna Levinsohn Tavor, Yifat Wiener, Stanislav Kotcherov, Amit Haboosheh, Ron Maymon

**Affiliations:** 1 Department of Obstetrics and Gynecology, Shamir (Assaf Harofeh) Medical Center, Zerifin, and Faculty of Medicine and Health Sciences, Tel Aviv University, Tel Aviv, Israel; 2 Department of Urology, Shamir (Assaf Harofeh) Medical Center, Zerifin, and Faculty of Medicine and Health Sciences, Tel Aviv University, Tel Aviv, Israel; 3 Department of Neuroradiology, Hadassah Medical Center, Jerusalem, Israel

**Keywords:** Fetal urinary ascites, Bladder rupture, Paraurethral cyst, Prenatal diagnosis

## Abstract

Fetal urinary ascites is an uncommon prenatal finding most often associated with lower urinary tract obstruction (LUTO). We report a rare case of fetal urinary ascites caused by bladder rupture secondary to suspected urethral compression by a paraurethral cyst. A 26-year-old gravida 3 para 1 woman was referred at 32 weeks’ gestation due to newly detected isolated fetal ascites and mild polyhydramnios. Detailed ultrasonography suggested bladder wall defect and a cystic structure adjacent to the right inguinal canal. Fetal MRI confirmed bladder rupture and supported the suspicion of LUTO. Conservative management was adopted due to stable fetal condition and preserved renal appearance. The ascites resolved spontaneously before delivery. A healthy female neonate was born at term, and the paraurethral cyst resolved spontaneously postnatally. This case highlights a rare etiology of fetal urinary ascites and demonstrates that expectant management may result in favorable outcomes in selected cases.

## What does this study adds to the clinical work

**Table:** 

Paraurethral cyst should be considered a rare cause of fetal urinary ascites and suspected lower urinary tract obstruction due to bladder rupture. In selected stable cases, careful imaging and multidisciplinary evaluation may support conservative management with favorable neonatal outcome.	

## Case presentation

A 26-year-old G3P1 woman with an unremarkable medical and obstetric history was referred at 32 weeks’ gestation due to newly detected isolated fetal ascites and mild polyhydramnios. Routine prenatal screening had been normal. Doppler evaluation excluded fetal anemia. Detailed ultrasonography demonstrated significant fetal ascites and a suspected defect in the bladder wall. A small cystic structure was identified adjacent to the right inguinal canal. Fetal MRI confirmed the presence of bladder rupture and supported the suspicion of lower urinary tract obstruction (LUTO), likely secondary to urethral compression by a paraurethral cyst.

Comprehensive genetic evaluation, including chromosomal microarray analysis and whole-exome sequencing, revealed no abnormalities. After multidisciplinary consultation involving obstetrics, pediatric urology, and radiology, conservative expectant management was recommended given the stable fetal condition and preserved renal appearance.

Follow-up ultrasound at 34 weeks demonstrated spontaneous resolution of the ascites. At 38 weeks, a healthy female neonate weighing 3270 g was delivered vaginally with Apgar scores of 8 and 9 at one and five minutes, respectively. Postnatal physical examination revealed a small paraurethral cyst, which resolved spontaneously within 48 h. Postnatal renal ultrasonography and voiding cystourethrogram confirmed normal urinary tract anatomy and function. At seven-week follow-up, the infant showed normal growth and renal function without recurrence.

This case highlights a rare cause of fetal urinary ascites due to bladder rupture from suspected paraurethral cyst obstruction and demonstrates that conservative management can lead to an excellent perinatal outcome in selected cases.

## Discussion

Prenatal paraurethral cysts represent an exceptionally rare etiology of fetal urinary ascites and suspected lower urinary tract obstruction (LUTO). This case underscores the importance of comprehensive imaging, multidisciplinary evaluation, and careful patient selection, as conservative management in stable fetuses can lead to spontaneous resolution and favorable neonatal outcomes while avoiding unnecessary invasive intervention [1–4].

**Fig. 1:**
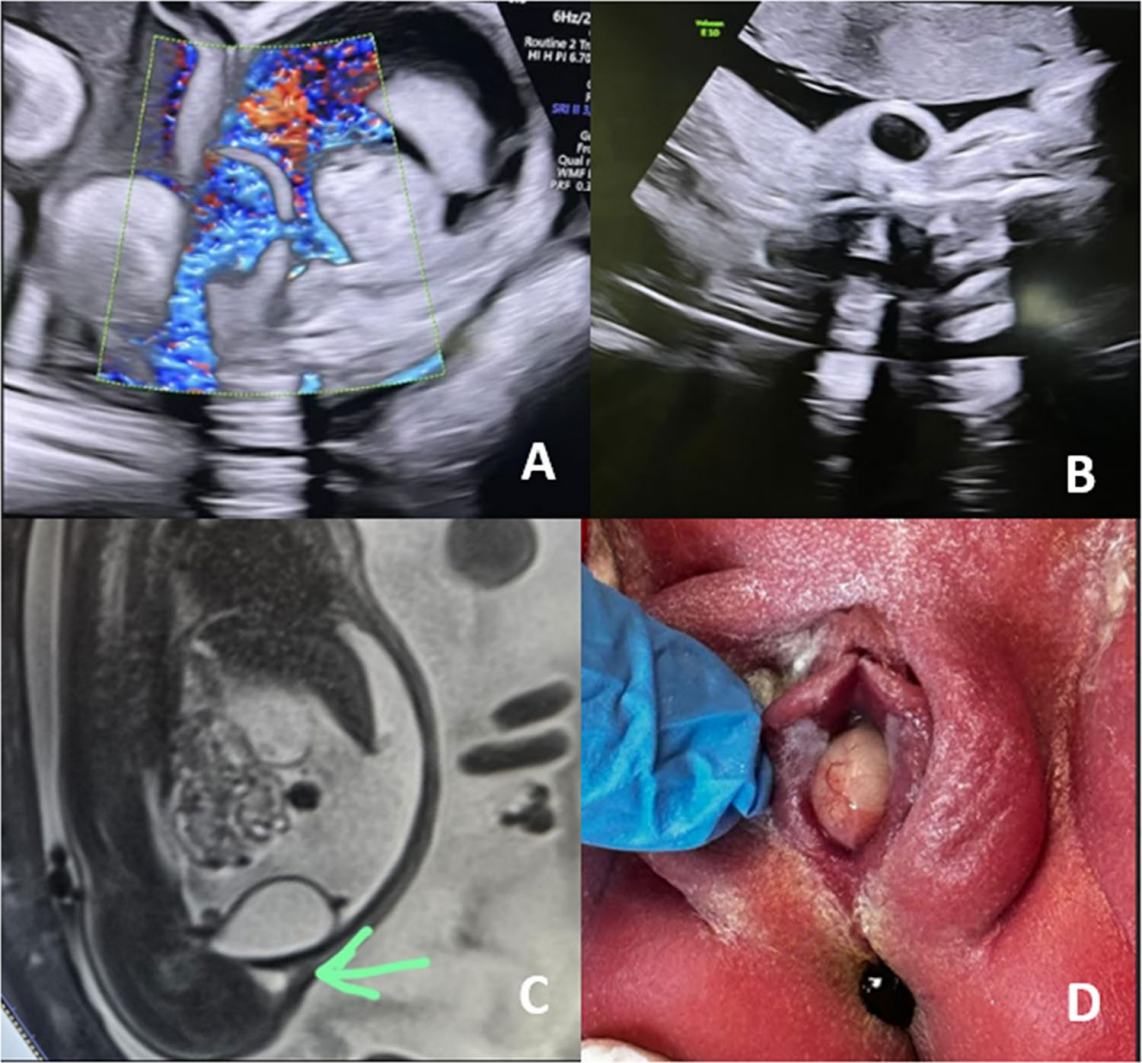
Prenatal and postnatal imaging of a paraurethral cyst complicated by bladder rupture. **A** Ultrasound with Doppler showing fluid flow from bladder and urinary ascites. **B** Cross-sectional view showing pelvic cyst lesion in the inguinal canal. **C** mid sagittal MRI view showing pelvic cyst lesion in the inguinal canal, **D** Postnatal appearance: cyst at vaginal introitus consistent with para ureteral cyst

## Data Availability

No datasets were generated or analysed during the current study.
